# Pituitary Apoplexy Associated with Endocrine Stimulation Test: Endocrine Stimulation Test, Treatment, and Outcome

**DOI:** 10.1155/2012/826901

**Published:** 2012-08-15

**Authors:** Takahiro Yamamoto, Shigetoshi Yano, Jun-ichiro Kuroda, Yu Hasegawa, Takuichiro Hide, Jun-ichi Kuratsu

**Affiliations:** Department of Neurosurgery, Faculty of Life Sciences, Kumamoto University School of Medicine, 1-1-1, Honjo, Kumamoto 861-8556, Japan

## Abstract

Pituitary apoplexy is a rare clinical syndrome attributable to hemorrhage or hemorrhagic infarction of pituitary tumors or pituitary glands. The features of pituitary apoplexy associated with the endocrine stimulation test remain to be elucidated and the importance of surgical treatment has not been discussed enough. We report two rare patients who were treated successfully by endoscopic endonasal transsphenoidal surgery within several hours after onset of pituitary apoplexy associated with the endocrine stimulation test. Their postoperative course was uneventful. We reviewed earlier reports on this clinical entity, document its features especially as related to the endocrine stimulation test, discuss the significance of immediate surgical treatment, and present our treatment outcomes. Performing only conservative treatment is not recommended. We suggest that the necessity of endocrine stimulation test should be assessed on a case-by-case basis and in patients subjected to the test, and neurosurgical support should be sought.

## 1. Introduction

Pituitary apoplexy is a life-threatening clinical syndrome thought to be attributable to hemorrhage or hemorrhagic infarction of pituitary tumors or pituitary glands [1]. Suggested precipitating factors are hypertension, anticoagulation, and bromocriptine therapy, and pregnancy [2–27]. There are few reports on the occurrence of pituitary apoplexy as a complication of the endocrine stimulation test, and its features remain to be elucidated. We report two rare patients with pituitary apoplexy associated with the endocrine stimulation test who were treated successfully by surgery and present a review of the literature. We also discussed about the necessity of neurosurgical support.

## 2. Case Presentation

### 2.1. Case 1

**Figure 1:**
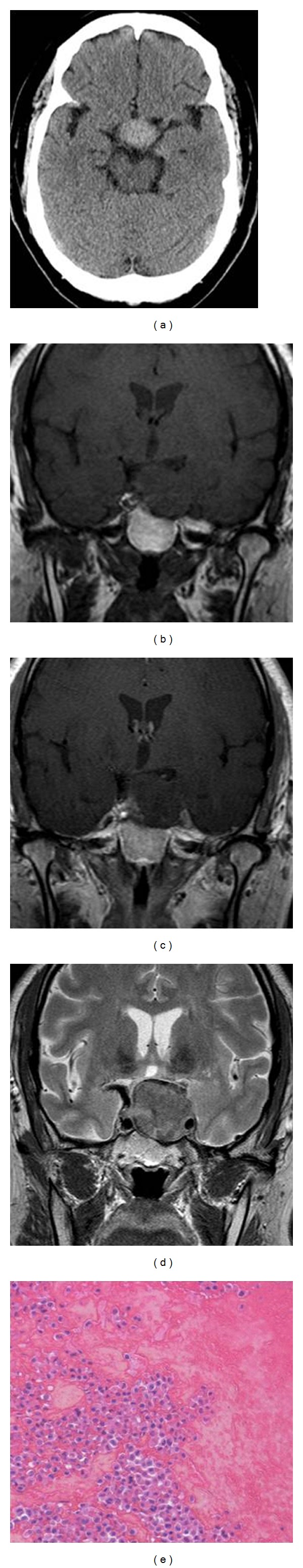
Case 1. CT scan performed 1 hr after onset demonstrates intratumoral hemorrhage (a), T1-weighted MRI showing a pituitary tumor extending into the suprasellar cistern (b), after onset gadolinium enhanced coronal T1-weighted MRI showing no enhancement of the pituitary tumor (c), T2-weighted MRI showing a pituitary tumor with a low-signal-intensity rim. This finding was suggestive of pituitary apoplexy (d), Specimen stained with hematoxylin and eosin. Hemorrhage and necrosis on the right side of the picture and area of nonnecrotic papillary-patterned tumor tissue on the left (e).

A 56-year-old woman with a one-year history of visual disturbance was admitted to our hospital for the evaluation of a suprasellar tumor. Physical examination at admission revealed visual disturbance (temporal hemianopia on the left side) but no other neurological disorders or endocrinological symptoms. Her baseline levels of pituitary hormones were normal. Computed tomography (CT) and magnetic resonance imaging (MRI) studies demonstrated a pituitary adenoma with suprasellar extension and superior displacement of the optic chiasm. To evaluate her hormonal responses we performed combined endocrine stimulation tests with growth-hormone-releasing hormone (GRH, 100 *μ*g), thyrotropin-releasing hormone (TRH, 250 *μ*g), luteinizing hormone-releasing hormone (LH-RH, 100 *μ*g), and corticotrophin-releasing hormone (CRH, 100 *μ*g). Fifteen minutes after the intravenous bolus injection she complained of severe headache, and this was followed by vomiting, progressive visual disturbance, and left oculomotor paralysis. She was alert but her symptoms gradually worsened. Emergency CT and MRI revealed intratumoral hemorrhage (Figures 1(a)–1(d)). Four hours after onset, her left visual acuity was reduced to total blindness; there was temporal hemianopia in the right visual field. At emergency endoscopic endonasal transsphenoidal surgery was (ETSS) performed 5 hr after onset. Partially, tumor was solid and reddish-brown, different from the typical feature of pituitary adenoma. The tumor was totally removed. Pathological examination revealed hemorrhagic and necrotic area in nonnecrotic papillary-patterned tumor tissue (Figure 1(e)). Postoperatively, her headache, nausea and left oculomotor paralysis resolved, and her vision returned to the preonset level. Because of her diabetes insipidus (DI) she received transient desmopressin replacement therapy. She was able to resume her normal life.

### 2.2. Case 2

**Figure 2:**
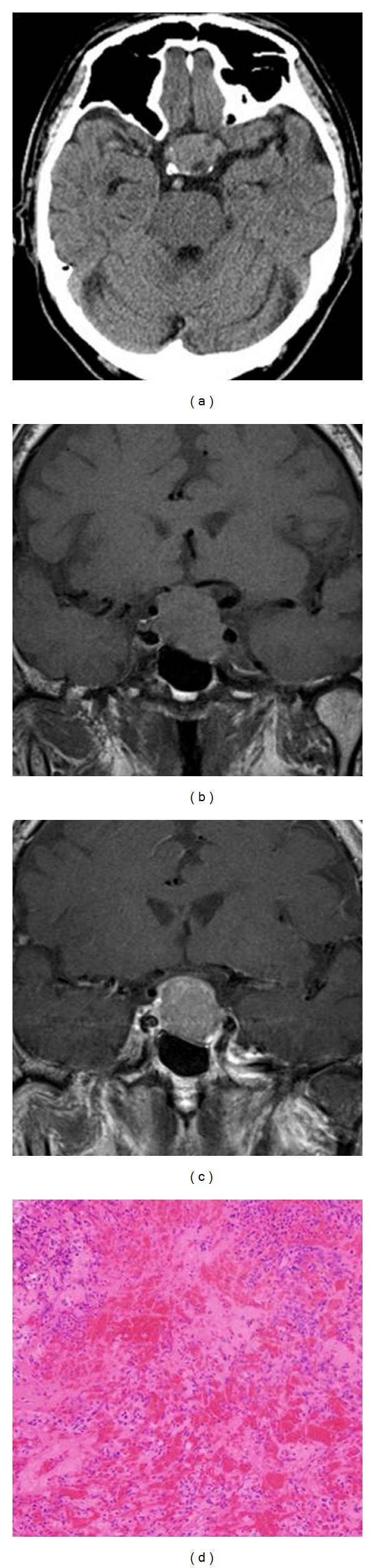
Case 2. CT scan performed 1 hour after onset demonstrates no evidence of intratumoral hemorrhage or acute enlargement of the tumor size (a), T1-weighted MRI performed after onset shows a pituitary tumor extending into the suprasellar cistern. There was no evidence of intratumoral hemorrhage or acute infarction (b), after onset gadolinium-enhanced T1-weighted MRI showing uniform enhancement of the pituitary tumor (c), and pathological examination revealed papillary-patterned adenoma with diffuse hemorrhage and necrosis (d).

A 73-year-old man with an 8-month history of visual disturbance was referred to our hospital. Five years earlier, when he was diagnosed with suprasellar tumor, he was asymptomatic and without visual disturbance. In the intervening five years, his tumor gradually enlarged and became symptomatic. At admission to our hospital, visual field examination revealed temporal hemianopia on the left side. He had no other neurological disorders or endocrinological symptoms. His baseline pituitary hormone levels were normal. CT and MRI demonstrated a pituitary adenoma with suprasellar extension and superior displacement of the optic chiasm. For preoperative endocrine evaluation we performed combined endocrine stimulation tests with growth hormone releasing peptide-2 (GHRP2, 100 *μ*g), TRH (250 *μ*g), LH-RH (100 *μ*g), and CRH (100 *μ*g). Twenty minutes after the intravenous bolus injection he complained of progressive visual disturbance. He was alert and experienced neither headache nor nausea. Emergency CT and MRI performed one hr after onset showed no evidence of intratumoral hemorrhage (Figures 2(a)–2(c)), two hr after onset his left visual acuity was reduced to total blindness. There was no visual disturbance on the right side. He underwent ETSS 5 hr after onset, and the tumor was totally removed. Same as Case 1, tumor was partially solid and reddish-brown. Pathological examination revealed papillary-patterned adenoma with diffuse hemorrhage and necrosis (Figure 2(d)). His postoperative course was uneventful. One month after the operation his visual disturbance resolved completely, and he required no hormone replacement therapy.

## 3. Discussion

Pituitary apoplexy is a rare clinical syndrome thought to be attributable to hemorrhage or hemorrhagic infarction of pituitary tumors or pituitary glands. It is characterized by the sudden onset of headache, vomiting, visual impairment, and decreased consciousness. Reported precipitating factors of pituitary apoplexy are hypertension, anticoagulation and bromocriptine therapy, pregnancy, and angiography [1, 22]. Although pituitary apoplexy associated with the endocrine stimulation test has been reported (Table 1), its features remain to be elucidated [2–27]. Of the 32 previously-reported patients and our two patients with pituitary adenomas who experienced pituitary apoplexy, 16 (47%) had nonfunctioning, 8 (23.5%) GH-secreting, 5 (14.7%) prolactin (PRL)-secreting, 3 (8.8%) follicle-stimulating hormone (FSH)-secreting, and 2 (5.9%) had adrenocorticotropic hormone (ACTH)-secreting adenomas. This proportion is similar to the frequency among the types of pituitary adenomas [28]. This suggests that there is no strong correlation between the types of pituitary adenoma and the elicitation of pituitary apoplexy by the endocrine stimulation test. On the other hand, there appears to be a relationship between the size of pituitary adenomas and pituitary apoplexy associated with the endocrine stimulation test. Our review of the literature showed that 93% of previouslyn reported patients manifested extrasellar extension of their pituitary tumors. TRH (26 cases, 76.4%) and LH-RH (23 cases, 67.6%) were the hormonal stimulants most commonly associated with the elicitation of pituitary apoplexy. Although the precise mechanisms of pituitary apoplexy associated with TRH- and LH-RH stimulation remain unclear, it has been suggested that TRH elevates the serum level of norepinephrine, and that vasospasm or pressor effects may be precipitating factors [3, 15, 29]. Others proposed that TRH directly activates the tumor cells, or that LH-RH stimulation increases metabolic activity leading to vascular accidents [6, 12, 15, 18, 29]. Interestingly, the number of hormonal stimulants used in the endocrine stimulation test has no bearing on the elicitation of pituitary apoplexy; the Spearman correlation was *ρ* = − 0.40 with a *P* value of 0.6 (Table 1(a)). Excluding the two patients reported here, the incidence of pituitary apoplexy among patients subjected to the endocrine stimulation test with four stimulants was 0 in our department. Because this test is convenient and the incidence of test-elicited pituitary apoplexy is low, it is used widely to investigate the reserve function of the pituitary in patients with pituitary adenoma. Nonetheless, based on earlier reports and our experience with the two patients reported here, we think that the necessity for performing the endocrine stimulation test must be evaluated on a case-by-case basis.

Among the 34 patients, detailed treatments and outcomes were available in 23 (Table 1(b)); 20 patients underwent surgery, and the other 3 were treated conservatively [3, 4, 7, 8, 11, 13–15, 17–23, 26, 27, 29]. Of the latter, all experienced symptom exacerbation after suffering pituitary apoplexy [7, 8, 14, 21, 26]. Among the 20 surgically treated patients, in 14 the symptoms worsened after the occurrence of pituitary apoplexy while in the other six they were unchanged or improved [3, 4, 11, 13, 15, 17–20, 22, 23, 27, 29]. In three patients who underwent tumor removal on the day of onset of pituitary apoplexy, the symptoms were worse after than before onset while in another four they were improved or unchanged [3, 11, 15, 18, 29]. In our cases, the patients underwent surgery within 5 hr of apoplexy onset, the symptoms attributable to pituitary apoplexy were improved. Because pituitary apoplexy associated with the above-cited precipitating factors is rare, controversy continues to surround the standard treatment [1]. In some instances the symptoms associated with precipitating factors improved when surgery was performed within 7-8 days of apoplexy onset [1, 30].

Our review of the literature suggests that patients with pituitary apoplexy elicited by the endocrine stimulation test should be operated within several hours after its onset, that is, sooner than patients with pituitary apoplexy associated with the other precipitating factors. Performing only conservative treatment is not recommended. We recommend that the necessity for endocrine stimulation tests should be considered carefully and that they be performed with neurosurgical support.

## Figures and Tables

**Table 1a:** (a) Reported cases of pituitary apoplexy associated with endocrine stimulation test: diagnosis, extension, and stimulation test

Case no.	Author	Year	Age/sex	Diagnosis	Extension	Stimulation test
1	Dunn et al.	1975	22/F	GH secreting	Uncertain	TRH, glucose, insulin
2	Silverman et al.	1978	31/M	PRL secreting	Extrasellar extension	Chlorpromazine
3	Jordan et al.	1979	21/F	ACTH secreting	Uncertain	Dexamethasone
4	Cimino et al.	1981	48/M	Nonfunctioning	Extrasellar extension	TRH, LH-RH
5	Drury et al.	1982	59/F	Nonfunctioning	Extrasellar extension	TRH, LH-RH, glucagon
6	Drury et al.	1982	66/M	GH secreting	Intrasellar	TRH
7	Drury et al.	1982	39/F	PRL secreting	Extrasellar extension	TRH, LH-RH
8	Drury et al.	1982	28/M	PRL secreting	Extrasellar extension	TRH, LH-RH
9	Bernstein et al.	1984	48/M	Nonfunctioning	Extrasellar extension	TRH, LH-RH, insulin
10	Korsic	1994	56/M	FSH secreting	Extrasellar extension	LH-RH
11	Chapman et al.	1979	39/F	PRL secreting	Extrasellar extension	TRH, LH-RH, insulin
12	Lever et al.	1986	19/F	GH secreting	Intrasellar	TRH
13	Shirataki et al.	1988	50/F	GH secreting	Extrasellar extension	Bromocriptine
14	Harvey et al.	1989	50/M	Nonfunctioning	Uncertain	Insulin
15	Arafah et al.	1990	41/F	PRL secreting	Extrasellar extension	LH-RH
16	Masson et al.	1993	54/F	FSH secreting	Extrasellar extension	LH-RH
17	Okuda et al.	1994	60/F	Nonfunctioning	Extrasellar extension	TRH, LH-RH, insulin
18	Vassallo et al.	1994	81/M	Nonfunctioning	Uncertain	TRH, LH-RH, L-Dopa
19	Masago et al.	1995	48/M	FSH secreting	Extrasellar extension	TRH, LH-RH, insulin
20	Masago et al.	1995	54/M	Nonfunctioning	Extrasellar extension	TRH, LH-RH
21	Szabolcs et al.	1997	54/M	Nonfunctioning	Extrasellar extension	TRH
22	Otsuka et al.	1998	31/F	GH secreting	Extrasellar extension	GRF, TRH, LH-RH, CRH
23	Dökmetaş et al.	1999	28/F	GH secreting	Extrasellar extension	TRH
24	Sanno et al.	1999	55/M	Nonfunctioning	Extrasellar extension	GRF, TRH, LH-RH, CRH
25	Lee et al.	2000	34/M	GH secreting	Extrasellar extension	TRH, LH-RH, insulin
26	Riedl et al.	2000	71/F	Nonfunctioning	Extrasellar extension	GRF, TRH, LH-RH, CRH
27	Matsuura et al.	2001	63/M	Nonfunctioning	Extrasellar extension	TRH, LH-RH, insulin
28	Rotman et al.	2003	19/F	ACTH secreting	Extrasellar extension	CRH
29	Yoshino et al.	2007	36/M	Nonfunctioning	Extrasellar extension	TRH, LH-RH, insulin
30	Yoshino et al.	2007	38/M	Nonfunctioning	Extrasellar extension	TRH, insulin
31	Wang et al.	2007	41/F	GH secreting	Extrasellar extension	TRH, LH-RH, insulin, L-Dopa
32	Kılıçlı et al.	2010	52/M	Nonfunctioning	Extrasellar extension	TRH, LH-RH, insulin
33	Our cases	2011	56/F	Nonfunctioning	Extrasellar extension	GRF, TRH, LH-RH, CRH
34	Our cases	2011	73/M	Nonfunctioning	Extrasellar extension	GHRP2, TRH, LH-RH, CRH

**Table 1b:** (b) Reported cases of pituitary apoplexy associated with endocrine stimulation test: treatment and outcomes of the 23 cases, for whom detailed treatments and outcomes were available

Case no.	Treatment	Interval from onset to surgery	Outcomes
1	Medication	—	GH reduction, DI
2	Craniotomy	Undocumented	Panhypopituitarism
3	Craniotomy	Undocumented	Visual disturbance, hemiparesis, aphasia
9	Transsphenoidal surgery	The same day (8.5 hours)	Visual disturbance, hemiparesis, aphasia
11	Craniotomy	3 days later	Recovered completely
13	Transsphenoidal surgery	11 days later	Recovered completely
14	Transsphenoidal surgery	Undocumented	Hypopituitarism
17	Craniotomy	The same day and 17 days after	Recovered completely
18	Medication	—	Hypopituitarism
19	Craniotomy	The same day (7 hours)	Recovered completely
20	Craniotomy	5 days later	Hypopituitarism
22	Transsphenoidal surgery	4 days later	Hypopituitarism
24	Transsphenoidal surgery	2 weeks later	Hypopituitarism
25	Transsphenoidal surgery	9 days later	Hypopituitarism
26	Transsphenoidal surgery	2 days later	Visual disturbance, ophthalmoplegia
27	Transsphenoidal surgery	1 day later (30 hours)	Improved to the level of preoperative state
28	Medication	—	Hypopituitarism
29	Transsphenoidal surgery	The same day and 21 days after	Hypopituitarism
30	Transsphenoidal surgery	7 days later	Hypopituitarism
31	Transsphenoidal surgery	2 days later	Ophthalmoplegia
32	Transsphenoidal surgery	The same day	Recovered completely
33	Transsphenoidal surgery	The same day	Visual disturbance
34	Transsphenoidal surgery	The same day	Recovered completely
